# Smartphones as a gateway to independence and adaptation: older Mandarin-speaking immigrants’ use of smartphones for everyday living

**DOI:** 10.1093/geroni/igag032

**Published:** 2026-04-15

**Authors:** Prince C Ekoh, Sepali Guruge, Jie Zhang, Kateryna Metersky, Denise S Cloutier, Hongmei Tong, Christine A Walsh

**Affiliations:** School of Social Work, Department of Social Sciences, University of New Brunswick, Saint John, New Brunswick, Canada; Dibịa Akwụkwọ, Social Solutions Research Group (SSRG), Nsukka, Nigeria; Daphne Cockwell School of Nursing, Toronto Metropolitan University, Toronto, Ontario, Canada; Gustavson School of Business, University of Victoria, Victoria, British Columbia, Canada; Institute on Aging and Lifelong Health (IALH), University of Victoria, Victoria, British Columbia, Canada; Daphne Cockwell School of Nursing, Toronto Metropolitan University, Toronto, Ontario, Canada; Department of Geography, University of Victoria, Victoria, British Columbia, Canada; Institute on Aging and Lifelong Health (IALH), University of Victoria, Victoria, British Columbia, Canada; Department of Social Work, MacEwan University, Edmonton, Alberta, Canada; Faculty of Social Work, University of Calgary, Calgary, Alberta, Canada

**Keywords:** Digital technology, Digital inclusion, Gerontechnology, Older immigrants

## Abstract

**Background and Objectives:**

The growing integration of digital technologies into everyday life has reshaped how older adults engage with their social environments, access information, and navigate health and community services. Yet, limited research has examined these dynamics among Mandarin-speaking older immigrants in Canada, a rapidly expanding population facing intersecting challenges of ageing, migration, and linguistic adaptation. Drawing on qualitative data from the national Inclusive Communities for Older Immigrants project, this study explores how Mandarin-speaking older immigrants use smartphones in their daily lives and how these technologies contribute to adaptation, independence, and well-being.

**Research Design and Methods:**

Data were obtained from 102 participants living in 7 cities across 4 provinces in Canada. The data were deductively and inductively coded with NVivo 15© and analyzed thematically.

**Results:**

Findings reveal 3 interconnected domains of smartphone use in support of daily living: (a) access to community resources, healthcare, home, and community safety; (b) play and leisure, including digital gaming, music, and online reading; (c) empowerment, navigating unfamiliar environments, enhanced autonomy, and reduced dependence on family members. Despite these benefits, participants reported persistent barriers to smartphone use, including linguistic inaccessibility, inconsistent translation services, and fears of digital dependence.

**Discussion and Implications:**

The study highlights the dual role of smartphones as both facilitators and barriers to digital inclusion, underscoring the need for culturally and linguistically responsive digital supports to promote equitable digital technological participation among Mandarin-speaking older immigrants in Canada.

**Innovation and Translational Significance**: This study is innovative in applying a sociotechnical ageing framework to examine smartphone use among older immigrants, a population that remains underrepresented in digital gerontology and migration scholarship. Rather than treating technology as a neutral tool or focusing solely on access and skills deficits, the study conceptualizes smartphones as sociotechnical infrastructures that actively shape social relations, belonging, and participation in later life. By centering Mandarin-speaking older immigrants across multiple Canadian cities, the research advances culturally grounded understandings of digital inclusion and challenges deficit-oriented narratives that frame older immigrants as technologically disengaged or socially isolated.

Over the past decade, the global proliferation of smartphones has transformed communication, mobility, and access to services. Older people have consistently constituted the largest population with limited access to smartphones and the internet (Zambianchi et al., 2019), accounting for approximately 94% of the digitally excluded population (Centre for Ageing Better, 2018). Although a persistent digital divide remains, recent evidence indicates a steady increase in internet and smartphone adoption among older adults globally (Eurostat, 2022; Government of Canada, 2025; Statistics Canada, 2021, 2023). In Canada, for example, internet use among adults aged 65 and over rose from 32% in 2007 to 68% in 2016, and by July 2020, amid the height of the COVID-19 pandemic, 80% reported daily internet use, and 65% owned a smartphone (Government of Canada, 2025). Nevertheless, uptake varies significantly by age cohort: while usage rates are relatively high among the “young old” (65-74), fewer than 50% of Canadians who are 80 years of age or older use the internet (Government of Canada, 2025). Comparable trends are observed in the United States, where 59% of adults aged 65–69 report smartphone use, compared with just 17% of those aged 80 and over (Anderson & Perrin, 2017).

Furthermore, multiple individual and structural factors shape smartphone use in later life. Higher income and education levels are associated with greater use of digital devices (Anderson & Perrin, 2017), while limited prior experience with digital devices is a barrier for many older adults (Fisk et al., 2009). Age-related challenges intersect with technology design issues; many smartphones were developed without considering older adults as major users, resulting in complex interfaces, small icons, and reduced readability, all of which impede adoption (Ekoh et al., 2023). Perceived utility can also influence engagement: For example, older adults often limit smartphone use to essential functions, such as maintaining social contacts, making emergency calls, and texting, showing they lack knowledge of the full capacities of smartphones (Ekoh et al., 2023, 2025).

The COVID-19 pandemic catalyzed a significant shift in smartphone use among older adults. Social distancing measures, reduced in-person service delivery, and the rapid expansion of virtual care prompted many older adults to adopt or increase their use of digital tools (Ekoh et al., 2021; Rorai & Perry, 2020; Xie et al., 2020). This shift accelerated access to telehealth, ehealth, and mhealth services, defined by the World Health Organisation (2021) as health and public health services delivered through mobile devices. Emerging evidence suggests that such technologies can enhance the quality of life of older adults by supporting fall detection, blood pressure and glucose monitoring, physical activity, and routine health checks (Marcolino et al., 2018; Wu et al., 2023). Smartphone use has also been associated with improved social participation (Ekoh et al., 2025), lower levels of depression (Briede-Westermeyer et al., 2020), and increased life satisfaction and happiness among older adults (Sagong & Yoon, 2018, 2022). Yet, concerns persist as excessive smartphone use has also been linked to smartphone addiction (Xu et al., 2023), cognitive strain, social withdrawal, and diminished well-being among community-dwelling older adults (Jeong et al., 2020; Kim et al., 2024; Nahas et al., 2018).

A growing body of literature documents the increasing integration of smartphones into everyday life among older adults in China, partly driven by the ubiquity of WeChat©, a multipurpose platform for communication, payments, information sharing, and social engagement (Cheng et al., 2024; Cui et al., 2024; Xu et al., 2023). Aligned with the theories of digital empowerment, which posit that digital technology can empower citizens based on functionality and implementation context (Goh et al., 2022; Sharma et al., 2022), studies have demonstrated that smartphone use among older Chinese adults facilitates mobility, reduces loneliness, enhances access to community resources (Liu et al., 2022), and is positively associated with life satisfaction (Cheng et al., 2024; Xu et al., 2023). In particular, research conducted during the COVID-19 pandemic revealed an increase in positive attitudes toward smartphone-based healthcare tools in China (Tse et al., 2023). Huang et al. (2023) found that ease of use and perceived opportunities for using smartphones are significantly shaped by older adults’ evaluations of smartphone utility. McKenna et al. (2025) underscored how older Chinese people use WeChat to navigate rapid societal changes driven by technological advancements while also resisting them.

Similarly, Smartphone use among older adults in Canada has expanded rapidly, particularly during the COVID-19 pandemic. Sixsmith et al. (2022) document increased reliance on smartphones among older adults to mitigate social isolation and loneliness during this period. Beyond social connectivity, research indicates that Canadian older adults use smartphones for a range of everyday functions, including caregiving coordination, participation in meaningful activities, physical activity monitoring, financial management, and health management (Freeman et al., 2022; Guruge et al., 2025; Metersky et al., 2025; National Institute on Ageing, 2024). Health-related smartphone use is especially prevalent: Sproul et al. (2023) report that 94.2% of older Canadians use health-related applications, with higher uptake among those aged 60–64 than among those aged 80 and over, highlighting both widespread adoption and age-related variation in digital engagement. Notably, recent national data suggest that racialized older adults are among the highest users of smartphones in Canada, with 87% of non-White adults aged 65 and over reporting smartphone use compared to 78% of White older adults (National Institute on Ageing, 2024). However, this survey does not provide in-depth details on how smartphones are used by this population in their daily lives.

Despite these advancements, little is known about smartphone use among Chinese older adults living outside China, particularly those residing in Canada. The limited studies available focus primarily on the relationships among smartphone use, loneliness, and social isolation (Brual et al., 2023; Ekoh et al., 2025; Government of Canada, 2025; Lin et al., 2023) and address infodemic [excessive spread of unreliable information] (Balyasnikova, 2024), leaving significant gaps in understanding how smartphones support daily activities, service navigation, and independent living. This is critical as smartphone use among older immigrants in Canada is consequential, as digital platforms increasingly mediate access to healthcare, social services, transportation, and civic services. Addressing this gap in the literature is crucial for advancing digital inclusion policies and ensuring that older Chinese immigrants, one of the largest populations of older immigrants in Canada, benefit equitably from technological innovations. Hence, guided by the question, ‘How do Mandarin-speaking older immigrants in Canada use smartphones in their everyday lives?’, our study examined this group’s smartphone use in daily life. As public services and community resources shift toward digital-first delivery models, older immigrants who rely heavily on smartphones, often their primary or sole digital device, risk being rendered invisible in digital inclusion policies that assume homogeneous patterns of technology use. Without empirical insight into how Mandarin-speaking older adults actually integrate smartphones into their everyday lives, policy and programmatic interventions may inadvertently reproduce exclusion rather than alleviate it.

## Method

This study is one component of a broader multi-phase initiative, Inclusive Communities for Older Immigrants (ICOI), which aims to explore the diverse factors contributing to social isolation among older immigrants and to develop culturally grounded strategies to improve their social connectedness. The larger ICOI initiative focuses on the three largest immigrant groups in Canada—Mandarin-speaking (East Asian), Punjabi-speaking (South Asian), and Arabic-speaking (Middle Eastern) older adults. Data were collected across seven cities in four provinces—Montreal (Quebec), Toronto and London (Ontario), Calgary and Edmonton (Alberta), and Vancouver and Victoria (British Columbia)—providing substantial geographic and demographic variation. However, this paper analyses how older Mandarin-speaking immigrants use smartphones in their daily lives, as this behavior was highly prevalent among this population.

This qualitative component of the study was guided by a critical ethnographic design, incorporating in-depth interviews and participant observation to examine the structural, cultural, and relational conditions shaping older immigrants’ everyday lives and experiences of social inclusion in Canada. Critical ethnography is particularly well suited to this inquiry, as it centers participants’ lived experiences while simultaneously interrogating the broader socio-political, institutional, and power structures that influence their opportunities, constraints, and forms of belonging. At the same time, the analysis was informed by an interpretive, qualitative orientation that foregrounds meaning-making, subjectivity, and social context. This interpretive lens facilitated a deeper understanding of how older immigrants themselves make sense of their experiences, relationships, and practices in everyday life. Together, critical ethnography and interpretive inquiry provided a complementary framework: the former illuminated structural and power-laden conditions shaping inclusion and exclusion, while the latter captured the subjective meanings through which older immigrants navigate, negotiate, and respond to these conditions.

A total of 102 participants were recruited in collaboration with immigrant-serving organizations, cultural associations, and community agencies across the seven study sites. Eligibility criteria required individuals to be aged 60 or older, born outside of Canada, self-identify as Mandarin-speaking, residing in one of the designated cities for at least one year at the time of data collection, and able to provide informed consent. Our recruitment strategy included distributing informational flyers, giving community presentations, relying on word-of-mouth referrals, and engaging directly at community gatherings. This multi-pronged approach supported the inclusion of participants with diverse settlement histories and levels of community engagement. The national research team collectively developed the interview protocol, and the questions covered participants’ daily living experiences. Examples of such questions include: “Tell me about your typical day,” and “What factors affect your ability to go out in your community?” Data collection took place in familiar community settings to enhance participant comfort and accessibility. Interviews lasted for 30 to 60 minutes and were conducted by bilingual and bicultural research assistants trained in qualitative interviewing. Participants selected whether they preferred to be interviewed in Mandarin or English. With consent, interviews were audio-recorded, translated when required, and transcribed verbatim to ensure accuracy.

All research assistants received training on study objectives, ethical considerations, confidentiality, and safe recruitment practices from the research leads prior to entering the field. Biweekly debriefing meetings with the national research lead and team members facilitated ongoing reflection, provided support, and allowed for continuous refinement of fieldwork strategies. Ethical approval for the study was granted by institutional review boards in each participating city.

A rigorous, multi-stage analytic strategy was employed, drawing on both deductive and inductive logics. Qualitative data were managed and organized using NVivo 15©. The national research team developed an initial codebook informed by the broader project’s quantitative findings, the relevant literature, and the emerging codes, which served as an initial analytical guide. This was followed by three local research teams engaging in open, inductive coding to capture context-specific nuances and emergent insights. Coding proceeded hierarchically, with top-level “grandparent” codes branching into progressively refined parent and child categories to reflect the complexity of participants’ accounts. The coding structure was revisited iteratively to ensure conceptual clarity and analytic depth. In line with Braun and Clarke’s (2023) reflexive thematic principles, coded segments were then systematically reviewed and synthesized into candidate themes, which were examined by the research team and consolidated, expanded, or rearticulated based on the robustness of the supporting data and their relevance to the study’s aims. Throughout the process, analytic credibility was strengthened through regular consultations with bilingual members of the research team, who provided linguistic and cultural insight into the data. This internal member-validation helped ensure that the themes faithfully represented participants’ meanings and experiences while maintaining conceptual coherence across the dataset. Our results were reported in themes and supported with relevant quotes. The quotes included labels consisting of participant numbers, city, age, gender (F=female, M=male), and immigration status (C=citizen, P=permanent resident, I=temporal immigrants). For example, the label “Participant 4, London, 68, F, C” represents a 68-year-old female participant from London who is a citizen and was the fourth participant interviewed.

## Findings

### Participants’ demographics

As presented in Table 1, the study sample comprised 102 Mandarin-speaking older adults from six Canadian cities, reflecting diversity in gender, age, migration history, and socioeconomic background. Participants ranged in age from 60 to 92 years and included 58 women and 44 men, with lengths of residence in Canada spanning one to 57 years. The majority held permanent resident or Canadian citizenship status, were married or widowed, and were fully retired. Educational attainment was generally high, with most participants reporting post-secondary qualifications, although levels varied across the sample.

**Table 1:** Demographic characteristics of participants.

Characteristic	Category	*n*
**Gender**	Female	58
	Male	44
**Age range**	60–92 years	—
**Years in Canada**	1–57 years	—
**Immigration status**	Immigrant	8
	Permanent resident	58
	Citizen	36
**Education level**	Bachelor’s degree	25
	Diploma	20
	Completed secondary education	18
	Some secondary education	14
	Master’s degree	8
	Primary education	2
	Other	15
**Marital status**	Married	63
	Widowed	27
	Divorced	7
	Separated	5
**Employment status**	Fully retired	80
	Partially retired	5
	Employed	7

### Themes and subthemes

**Figure 1:**
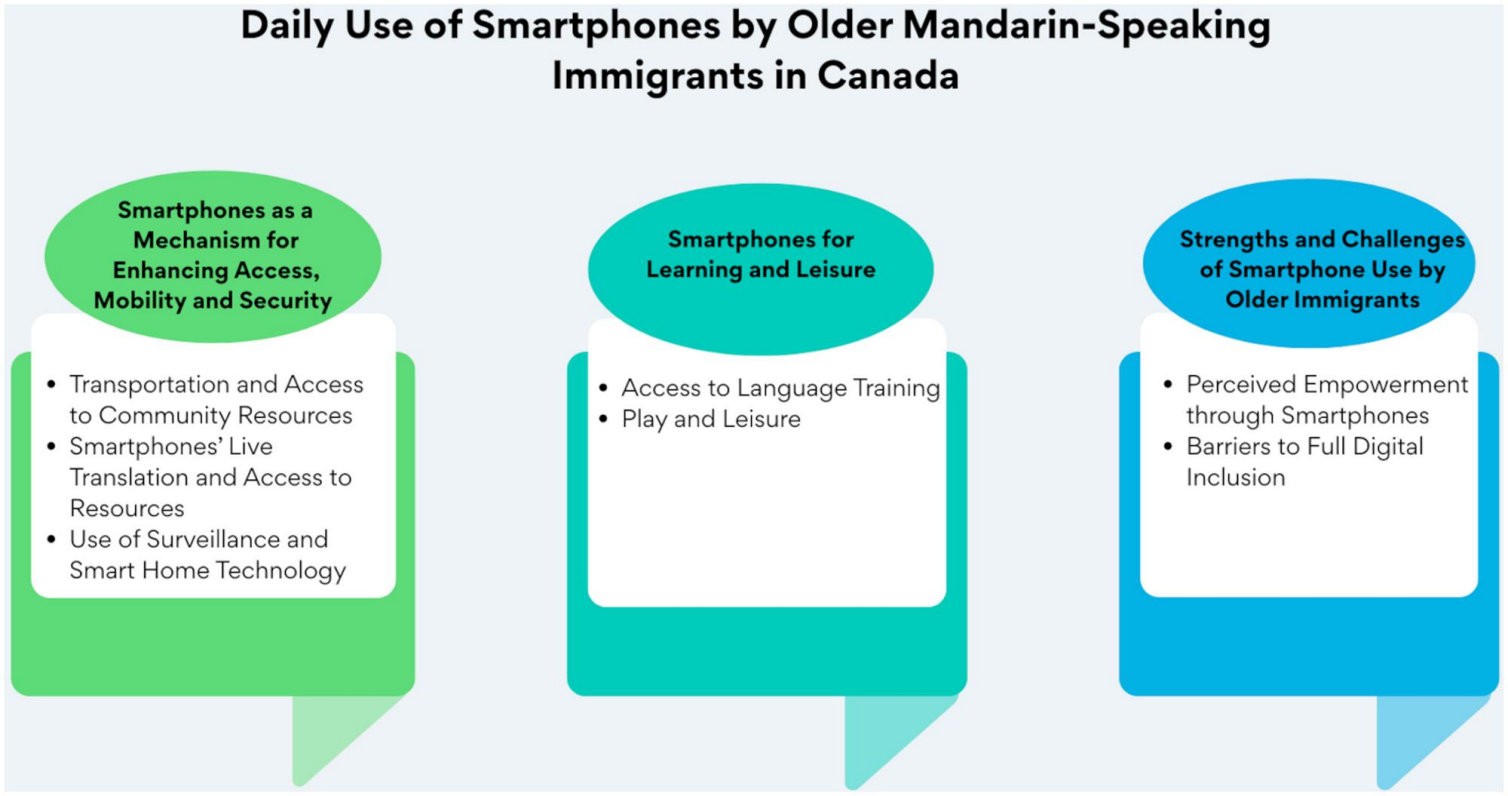
Themes and sub-themes demonstrating daily use of smartphones.

As shown in Figure 1, our analysis generated three overarching themes comprising six interrelated sub-themes. The first theme elucidates how smartphones facilitate access, mobility, and safety for Mandarin-speaking older immigrants, enabling them to navigate their environments with greater confidence. The second theme examines how smartphones function as essential tools for both leisure and lifelong learning, broadening opportunities for education. The final theme considers how smartphone use enhances older immigrants’ capacity for independent or semi-independent functioning, while simultaneously highlighting the persistent structural and linguistic barriers that limit their full digital inclusion.

### Smartphones as a mechanism for enhancing access, mobility, and security

Smartphones emerged as a critical technological resource, substantially enhancing the daily functioning and community integration of Mandarin-speaking older immigrants. This theme highlights how Mandarin-speaking older immigrants leveraged digital technologies to overcome structural, linguistic, and mobility-related barriers.

#### Transportation and access to community resources

Participants consistently reported that smartphones played a central role in enabling independent mobility. Through applications such as Google Maps© for transit planning, older immigrants were able to organize trips, examine route options, check schedules, and anticipate transit changes prior to leaving home. This form of pre-planning reduced uncertainty and fostered a sense of control over their movements. The use of smartphones to plan mobility was more prevalent amongst immigrants and permanent residents compared to citizens. This may be because they are newer to the country compared to citizens; hence, they may have less knowledge of the environment and face greater language barriers. As one participant explained: “On occasions when I take the bus, I try to plan my journey in advance. I check the bus routes and schedules to avoid any confusion” (Participant 9, London, 60, F, I). Another participant added:

If we must travel far, we will drive and utilise Google GPS. We used this method to travel by car for two weeks in Canada. We were able to book a camping ground, and once we arrive, we will use the app to translate and communicate with people there. I met a Chinese staff member at the camp, and we used the same camp on the way home. However, the Chinese staff were away, so we used the App to communicate. The staff member asked me for the details about the app and said he would share them with his colleagues. (Participant 8, Montreal, 68, F, P)

Aligned with the digital empowerment theories, smartphones enable older Mandarin immigrants, especially newcomers, to navigate their communities without relying on family and friends. Another permanent resident described how they use Google Maps to move around, irrespective of the mode of transport:

I walk and ride bikes. I used to drive around the city, but I don’t drive anymore. I also use public transportation, such as the bus… We can Google the bus schedule online before going out, or we can go out a bit earlier than the scheduled time, since we are both retired and have more free time than people who work. I prefer to wait for the bus at the bus stop rather than at home, because I can enjoy the fresh air and sunshine outdoors. (Participant 24, London, 72, M, P)

Beyond mobility, smartphones functioned as gateways to community-based resources such as grocery stores, libraries, and community centers. These spaces offered not only material support but also opportunities for social connection, especially with other Mandarin-speaking older immigrants. Participants described using online information to stay informed about local programming, religious gatherings, and political or cultural developments, thereby maintaining social engagement and socio-cultural continuity. As one participant mentioned:

I can access information online, at the library, through radio news broadcasts, or when I attend church and listen to fellow brothers and sisters or our pastor’s sermons. I listen attentively because I believe that at my age, the experiences of our past lives can help me understand and interpret many words in the Bible. I think it’s very beneficial. Because there’s a Chinese saying, “Scholars don’t have to leave their doors to know everything.” Especially as we get older, it’s not easy to travel far. Now, I’m still okay because the Internet reaches everywhere, so I can learn about events in Canada, other provinces, foreign countries, and different continents. So, I’m generally interested in politics, economics, culture, society, and education. (Participant 4, Vancouver, 72, M, C)

#### Smartphones’ live translation and access to resources

As mentioned in earlier language barriers are well-documented obstacles to healthcare access among older immigrants, and participants in this study echoed these challenges. Smartphones, particularly their real-time translation features, were frequently used to interpret forms, clarify medical instructions, communicate with healthcare staff, and schedule appointments. Smartphone translation features were found to be critical for helping participants access healthcare resources. While participants from different cities across Canada reported this, it was more prevalent among immigrants and permanent residents in Montreal. This prevalent use of translation apps by older immigrants in the intersection of being a newer immigrant and living in a French province may be due to the prioritization of French in the city of Montreal and to a recent policy that allows healthcare practitioners to refuse service to residents who have lived in Quebec for more than 6 months and lack French proficiency (Riga, 2025). Hence, some participants in Quebec reported using their smartphones to access telehealth to avoid these systemic barriers to healthcare. Two participants narrated:

There’s nothing we can do about the language problem because your mother tongue has been growing up since childhood, so you must not be good at English. I bought a translator, then I decided to use it on my mobile phone as well. So if I don’t understand, the translator will help me. I go to see a doctor with it wherever I go. When I see a doctor, I also use a translator; therefore, I can now talk to many doctors. For the intake form, I’ll translate it and fill it out myself. It’s great. I can do mobile translation, and that on my mobile phone, because I don’t want to trouble my children. Also, I believe people need to have a sense of self-confidence. (Participant 8, Calgary, 76, F, P)

It takes a long time to get the medical care needed, and it takes a long time to find volunteer translators. Therefore, I must figure out how to use the translate function on the phone for different daily encounters. I have a Huawei© cell phone that has a pre-installed Microsoft© translation app. (Participant 8, Montreal, 68, F, P)

Interestingly, smartphones were not only tools for receiving care but also platforms through which some Mandarin-speaking older immigrants provided health-related support to others in their linguistic community, especially for those who encounter language barriers in mainstream medical services. For example, one participant, a retired medical doctor, described using WeChat groups to disseminate health information and deliver online lectures, which started during the pandemic:

During the epidemic, they knew I was a doctor and invited me to manage a WeChat group [for health issues]… I thought it was not easy for everyone at that time … It was not easy for them to see a doctor. Most Chinese immigrants don’t understand the language … I had this sense of mission after I entered this group since I was a doctor … I would invite some colleagues I knew, whether they resided in local or foreign countries, mainly foreign, to join the WeChat group to answer some of the people’s questions … They asked me to continue to form a WeChat group because I like Tai Chi [a Chinese mind-body practice focused on improving balance and strength through movement and meditation]. After I participated in the online activities, I insisted on taking everyone with me to learn and practice Baduanjin [a Chinese exercise involving flowing movement, meditation, and breathing to improve health and balance] and Tai Chi online … and sometimes I also gave online health lectures… There are fewer people after the pandemic. However, I still insist on learning some health knowledge … I now give medical lectures, all voluntary. Even though I am retired, I still want to promote lifestyle medicine. In this regard, I am still fulfilling my responsibility so that everyone can live happily and for a long time in their later years. (Participant 9, Montreal, 72, F, C)

Another participant describes how she and her husband, also a medical doctor, encourage people to use telehealth:

One of my biggest concerns is this problem of seeking medical treatment. I think it’s the Quebec health care system. Well, it’s really inconvenient. If a person is really sick, I think it is very difficult to see a doctor. Now, telehealth is very popular; they all provide online consultations. He [my husband] is enthusiastic about those new innovative ways of seeing a doctor. My husband is keen to see his patients in person, consult people online, and participate in some of their discussions. (Participant 1, Montreal, 66, F, P)

Beyond using smartphones’ interpretation feature to facilitate access to physical health care, our participants and their family members also used smartphones to access psychosocial support, as evidenced in their responses to the interview questions. Particularly, one participant shared:

He (the participant’s husband) contacted an online psychological counsellor for help. My husband had a psychological consultation call. The second time he called, the conversation was interrupted by something at home. At that time, a friend happened to stop by our house to help us with computer issues related to online banking. My husband said we should postpone the conversation for a while, but he didn’t call [the psychological counsellor] again. (Participant 10, Edmonton, 76, F, C)

Participants also used smartphone translation to interpret public notices, workplace announcements, and road signs. When translation software yielded unclear results, they supplemented their understanding by sending photographs to family members or peers for further interpretation, demonstrating a hybrid use of digital and social capital. As one participant shared:

Sometimes, the manager would post a notice on your door. Sometimes, I can read it through a dictionary. If I don’t know how to read it, I will take a photo with my phone and send it to my daughter. Then they will tell me what I may need to do. (Participant 10, Montreal, 80, F, P)

However, while smartphones empower older Mandarin-speaking immigrants to access services independently, participants simultaneously expressed ambivalence about the reliability of translation technology. Many raised concerns about inaccurate translations, particularly in clinical settings where errors could have significant health implications. As an example, one participant outlined: “If we need to see a doctor, many medical terms are specialized, and Google Translate© makes mistakes, which might cause misdiagnosis. That is why I want to have translators hired for these essential aspects” (Montreal, Participant 8, 68, F, P). Other participants indicated that some care providers do not accept the use of smartphone translators, denying them that sense of independence in accessing services. A participant added, “It took me a while to be seen by a walk-in clinic doctor, and they required me to have a translator as they felt the app translator was inaccurate. I had to get a friend on the phone who was in the meeting to help me translate” (Participant 4, Vancouver, 72, M, C).

#### Use of surveillance and smart home technology

Smartphones also played an essential role in enhancing perceptions of safety and security—domains intimately tied to older adults’ quality of life. Participants discussed smartphones as the integrating hub that ties together cameras, sensors, lights, and family support: As one participant shared, “In addition to the camera, I also have light-sensing lights outside that will automatically turn on when someone comes over. The light will scare them away. They’ll think the homeowner turned on the lights” (Participant 4, Edmonton, 71, M, C). Another participant narrated how they use their surveillance camera:

I remembered our garage door was closed. But then he may have accidentally touched the button, and the door was opened again. Two days later, my daughter saw someone knocking on my door through the surveillance camera. She said someone knocked on our door twice. I said there must be something going on. I looked at the surveillance and found that the person knocking on the door was our neighbour…My daughter paid attention to the monitoring and talked to him when he knocked on the door for the third time. The neighbour told my daughter that your garage door has been open for three days. I was shocked. How come the garage door has been open for three days? The child must have mistakenly pressed the button when he went out and then opened the door again. (Participant 2, Edmonton, 77, F, P)

At the community level, participants reported using WeChat or WhatsApp© group chats to share safety alerts and collectively monitor neighborhood incidents. As one participant discussed,

We have a group chat where people can share information. In this case, this person shared this information and told us to keep an eye on suspicious people around our neighbourhood. If things happen, don’t hesitate to call the police. (Participant 34, London, 60, M, I)

This shows how smartphones help Mandarin-speaking older immigrants protect their homes directly and through community efforts. It reflects how digital communication reinforces community-based protective networks among immigrant older adults.

### Smartphones for learning and leisure

Smartphones were identified as critical digital tools that facilitated both educational engagement and leisure participation among Mandarin-speaking older immigrants. Smartphones supported psychosocial well-being, cultural continuity, and adaptive learning in later life.

#### Access to language training

Although translation functions enable newcomers to navigate their environments, participants underscored that genuine language proficiency in their host country remains essential for meaningful integration, independent mobility, and confident engagement with Canadian society. Smartphones provided an accessible platform for language learning, enabling newer Mandarin-speaking older immigrants to participate in formal and informal language training from home. Hence, many immigrants and permanent residents express appreciation for the flexibility smartphones provide them to learn the dominant language while balancing competing interests and responsibilities at home. One participant described shifting from community-based classes to self-directed online learning facilitated by smartphone technologies and social networks developed in the process:

I studied language at the Chinese Community Centre in my first year here. That year, I participated in the activities of the Chinese Community Centre to celebrate the National Day and the Spring Festival. Later, I left and felt that it was not ideal to study English there. Then I studied English by myself at home. I learn English at home. I learn from the Internet. When I made friends while studying, I kept all my contact information. Later, these friends formed a group. (Participant 2, Edmonton, 77, F, P)

#### Play and leisure

Leisure activities are increasingly recognized as central to older adults’ wellbeing, contributing to cognitive stimulation, emotional regulation, and social connectedness. Smartphones enabled participants to engage in culturally meaningful and recreational activities, including play online mahjong [a popular Chinese tile game] with their friends “I also like to play mahjong online, and I have a lot of group chats. I chat with my friends or learn about the news on my phone. This can also take me more than two hours” as well as mainstream games like chess and Sudoku [a logic-based number placement puzzle played on a 9x9 grid] (Participant 4, Toronto, 83, F, P). The participant added:

I am very busy every day, and I like to play. I will play on my mobile phone and computer, including games. I also like Sudoku; I bought a lot of Sudoku and spend over two hours daily on it. I may draw grids and create my own Sudoku. (Participant 4, Toronto, 83, F, P)

Beyond gaming, smartphones enabled access to digital reading, online singing classes, and other forms of virtual leisure, underscoring their role in expanding the recreational landscape for Mandarin-speaking older immigrants. As one participant explained:

At home, I cook twice a day, once around nine o’clock in the morning and the other at 4:30 in the afternoon, eating two meals a day. In the evening, I began playing games and sometimes listened to novels downloaded from the Internet, reading one or two paragraphs per day. What’s more, I can download songs from the Internet, and I love to listen to music because I have a singing class every week. (Participant 8, Toronto, 84, M, P)

These accounts demonstrate how digital leisure activities not only fill time but also contribute to continuity of interests, cultural linkage, and emotional well-being.

### Strengths and challenges of smartphone use by older immigrants

Our findings show that Mandarin-speaking older immigrants perceive the use of smartphones as empowering, improving their adaptation to their environment and independence. However, they also highlighted some barriers to smartphone use, including language barriers and concerns about banning smartphone apps such as WeChat.

#### Perceived empowerment through smartphones

Anchored on the theories of digital empowerment, smartphones also facilitated psychological empowerment by enabling participants to navigate daily challenges with greater confidence, while reducing their dependence on family members. While acknowledging the limitations of translation applications, participants expressed a sense of agency associated with their ability to “figure things out” independently. As one participant outlined,

I do use Google Translate, but it is not reliable. They can only assist with simple tasks, not complicated ones. My friends like to depend on their children, but I prefer to figure out what to do myself. As I have a son and my friends have daughters, their daughters seem more willing to help out. My son is very busy with his family, and their children must attend school. If there is anything broken in the apartment, I used Google Translate, but it didn’t work well because I put Chinese into English. However, when I translated English back to Chinese, it turned out to be different from what I put in Mandarin. (Participant 6, Montreal, 70, F, P)

Smartphones provided a form of “digital safety net,” encouraging Mandarin-speaking older adults to explore their neighborhoods and access community resources while maintaining a lifeline to family in case of difficulty. As one participant noted:

My daughter bought me a smartphone because my English isn’t very good, so whenever I get lost or can’t find my way, I call her for help. I generally don’t use public transport; for closer destinations, I prefer walking, and for farther places, I rely on my daughter to drive me there. (Participant 8, London, 75, F, P)

Thus, smartphone access served as an enabling tool for mobility, autonomy, and risk mitigation, which are key domains in immigrant adaptation.

#### Barriers to full digital inclusion

Despite these benefits, multiple linguistic, social, and systemic factors constrained full digital inclusion. Language remained the most pervasive barrier, as Mandarin-speaking older immigrants found both English and French interfaces challenging, even with translation applications. Hence, many participants, especially permanent residents, still recommend policy efforts to ensure human interpretation, indicating that while the use of smartphones can be empowering and improve independence, it cannot be a substitute for human interpretation support. A participant described the implications for health access:

I understand that there is a need for more labour in Canada due to an ageing population. However, the government should consider providing translation services in specialized departments, such as 911 [emergency services]. I tried using 811, then pressed 3 to book an appointment with a doctor or nurse. However, they only provide services in French, reducing the likelihood that older people with no language skills can access them. As for me, I tried to use Google Translate to understand the process of filling out a form. I can figure out what to do on my own. However, this situation increases my anxiety level and sometimes affects my sleep. (Participant 8, Montreal, 68, F, P)

Participants strongly advocated for Mandarin-specific support systems, such as telephone interpretation lines for healthcare settings, emphasizing that late-life language acquisition is difficult and easily lost without constant practice. As one participant pondered:

Can a telephone translation hotline be established and designated for Mandarin-speaking people to support the Chinese population in Montreal? It is a small investment to facilitate the integration of different cultures in Canada. The language barrier is very challenging, as there are few opportunities for older people to learn a language, especially in later life. It is also hard to maintain a new language, especially for older people. A specially designated telephone line for medical care could benefit older Chinese people. (Participant 5, Montreal, 67, M, P)

Concerns also emerged regarding digital over-reliance, potential smartphone addiction, and fears surrounding possible restrictions on WeChat, an essential communication tool for many older Chinese immigrants. Hence, there is a need for a balanced approach to smartphones as a supportive tool rather than a perfect solution to the diverse needs of older immigrants. A participant revealed that while smartphones make life easier, they lead to over-dependence and addiction. As one participant discussed,

However, we can also see people becoming addicted to the app, spending hours and hours on it. The development of social media platforms is a sign of technological advancement, but it also comes with a shortfall. (Participant 3, Victoria, 69, M, C)

While a few mentioned that there might be restrictions around the use of WeChat in Canada:

“I mostly use WeChat, though I heard the Canadian government might ban it” (Participant 19, London, 83, F, C). All these raise concerns among Mandarin-speaking older immigrants about the full adoption of digital technology in everyday life.

## Discussion

This national, multi-site qualitative study provides one of the first comprehensive examinations of how Mandarin-speaking older immigrants in Canada integrate smartphones into their everyday lives, revealing both the transformative potential of digital technologies and the persistent inequities that constrain full digital inclusion. Consistent with literature highlighting the growing uptake of digital tools among older adults (Metersky et al., 2025; Statistics Canada, 2023), our findings demonstrate that smartphones have become essential instruments for mobility, health navigation, communication, and psychosocial well-being among Mandarin-speaking older immigrants. However, the ways in which these technologies are adopted—and the challenges that accompany their use—remain deeply shaped by linguistic, socio-cultural, and structural factors.

A central contribution of our study is the documentation of how smartphones function as critical enablers of spatial and social mobility. Participants described using mapping applications, transit information, and digital trip-planning tools to navigate unfamiliar urban spaces with greater independence, expanding or maintaining their worlds rather than contracting them as often happens with migration. While the participants in our study do not use a super app, such as WeChat, these findings echo earlier studies in China demonstrating that smartphones help older adults overcome geographic barriers and maintain and expand their access to community resources (Cui et al., 2024; Liu et al., 2022; Xu et al., 2023). Within the Canadian context, where newcomers must adapt to dispersed services, unfamiliar transit systems, and limited language supports, smartphones appear to serve as compensatory tools that substantially reduce reliance on family members. This is particularly significant in immigrant contexts where intergenerational households often experience competing responsibilities and limited time for caregiving; hence, smartphones empower older immigrants to navigate their environment with little to no support.

Our study also deepens current understandings of the role of smartphones in supporting health navigation. Consistent with research identifying language as a central barrier to healthcare access for older immigrants (Dempsey et al., 2009; United Nations High Commissioner for Refugees [UNHCR], 2019), participants relied heavily on translation applications to complete forms, attend appointments, communicate symptoms, and manage tele-health interactions. This aligns with prior findings that older adults in Canada and China increasingly use smartphones for health management and digital care (Marcolino et al., 2018; Sproul et al., 2023; Wu et al., 2023). While these findings align with digital empowerment theories, which support the independent functioning of older adults through smartphones, they also point to the limits of these technologies: participants repeatedly expressed concerns about the inaccuracy of translation applications, particularly for specialized medical terminology, heightening the risk of misunderstanding and potential misdiagnosis. These limitations were amplified in a province such as Quebec, where French-language healthcare requirements create additional layers of exclusion. In several cases, participants attempted to circumvent systemic linguistic barriers by using tele-health services outside their province (Riga, 2025). Such experiences highlight structural inequities in Canadian health systems and point to the urgent need for linguistically and culturally appropriate digital health supports for older immigrants.

Unlike previous studies on the use of smartphones by older adults in both Canada and China, which did not account for the use of smartphones for language training (Sproul et al., 2023; Wu et al., 2023), many participants used smartphones to access language-learning resources, supporting immersion and communication in their host society. Smartphones also support meaningful leisure activities, underscoring the multifaceted roles of technology in maintaining cultural continuity, fostering cognitive stimulation, and promoting emotional well-being. This reinforces emerging gerontological findings that digital leisure contributes to improved mental health, increased social participation, and reduced loneliness among older adults (Briede-Westermeyer et al., 2020; Sagong & Yoon, 2018).

Importantly, the findings also reveal a distinct dimension of perceived empowerment. Many participants described feeling more autonomous when able to navigate services, translate information, and manage daily tasks without relying on their adult children. This aligns with theories of digital empowerment (Goh et al., 2022; Sharma et al., 2022), which outline that technology can support agency and self-efficacy in later life. In the context of migration, in which older adults often experience a loss of status, disorientation, and heightened dependence, this sense of technological autonomy holds both psychosocial and practical significance.

Despite these benefits, our study highlights persistent barriers to digital inclusion. Linguistic limitations remain the most significant challenge, shaping the accuracy of translation tools, the usability of apps, and access to emergency or health helplines. Participants repeatedly emphasized the inadequacy of digital supports for non-English or non-French speakers, reinforcing prior research that immigrant older adults experience disproportionate digital marginalization (Ekoh et al., 2023). Moreover, some participants expressed concerns about over-reliance on smartphones, digital fatigue, or potential addiction, issues increasingly documented in the literature (Jeong et al., 2020; Xu et al., 2023). These findings point to a complex interplay between individual adaptation, technological access, and broader structural constraints. Smartphones clearly serve as essential tools that promote independence, community participation, and access to healthcare for Mandarin-speaking older immigrants. However, technology alone cannot eliminate linguistic, systemic, and institutional barriers. Equitable access will require concerted policy efforts, including the development of age- and language-inclusive digital infrastructure, accessible translation and emergency services, culturally tailored digital literacy programs, and collaboration among immigrant-serving agencies, older immigrants, and technology developers.

## Strengths, limitations, and future research

As the first national study to document smartphone use among Mandarin-speaking older immigrants in Canada, to the best of our knowledge, this research offers critical insights into the evolving digital landscape of ageing and migration. However, it is possible that the significantly higher level of education among our study participants affected their smartphone use, as reported in other studies (Anderson & Perrin, 2017). Future research should further examine smartphone use among older immigrants with limited educational attainment. Further research can also explore the role of family networks in digital learning and the potential implications of emerging technologies, such as AI-assisted translation, on the inclusion and well-being of older immigrants. Ultimately, addressing the digital divide for Mandarin-speaking older adults is not simply a matter of technological access but of social justice, equity, and meaningful participation in Canadian society. Hence, there is a need for more tailored apps and platforms, as well as research to inform the design of these apps and ensure compatibility with the needs of older immigrants.

## Conclusion

This study demonstrates that smartphones play a multifaceted role in the everyday lives of Mandarin-speaking older immigrants in Canada, functioning as tools for learning, communication, leisure, safety, and self-determination. At the same time, the findings underscore enduring structural and linguistic challenges that shape digital participation. To our knowledge, this is the first national qualitative account of smartphone use among Mandarin-speaking older immigrants in Canada, and it contributes to the growing scholarship on technology, ageing, and migration. The findings call for targeted policy and program interventions, including improved multilingual digital services, culturally tailored technology training, and recognition of immigrant-specific digital needs, to ensure that technological advancements meaningfully support the social and cultural well-being of older Chinese immigrants.

## Data Availability

The data underlying this article cannot be shared publicly due to privacy reasons. The data will be shared on a reasonable request to the corresponding author.
